# Une forme rare et fulminante de gastrite: la gastrite emphysémateuse

**DOI:** 10.11604/pamj.2016.24.301.7518

**Published:** 2016-08-09

**Authors:** Ammar Mahmoudi, Mezri Maâtouk

**Affiliations:** 1 Service de Chirurgie Générale et Digestive, CHU Fattouma Bourguiba de Monastir; 2 Service d’Imagerie Médicale, CHU Fattouma Bourguiba de Monastir

**Keywords:** Gastrite emphysémateuse, tomodensitométrie, antibiothérapie, chirurgie, Emphysematous gastritis, tomodensiometry, antibiotic therapy, surgery

## Image en médecine

La gastrite emphysémateuse, caractérisée par la présence de gaz dans la paroi gastrique, est due à une infection par des germes “producteurs de gaz”. Son pronostic est sombre avec une mortalité très élevée, imposant un diagnostic et un traitement précoces. Le tableau clinique n'est pas spécifique. Le diagnostic est souvent radiologique. La chirurgie est indiquée en urgence en cas de nécrose, de perforation, de péritonite ou de détérioration rapide de l'état du patient sous traitement médical. Nous rapportons le cas d'un homme de 79 ans, aux antécédents de maladie d'alzheimer depuis 3 ans, diabétique sous antidiabétiques oraux depuis 30 ans, qui s'est présenté aux urgences pour une douleur épigastrique évoluant depuis 48 heures. A l'examen, il présentait un état septique avec fièvre à 39,5º C et frissons avec tendance au collapsus. Il était polypnéique et il y avait une défense abdominale à l'étage sus-ombilical. Il existait une hyperleucocytose à 25 000/mm^3^, une CRP à 280 mg/l et une acidose métabolique. La tomodensitométrie abdominale (A,B,C) montrait du gaz dans la paroi gastrique et confirmait le diagnostic de gastrite emphysémateuse. Les axes vasculaires digestifs, en particulier le tronc coeliaque, étaient perméables. Il n'y avait pas d'aéoportie. Une antibiothérapie à large spectre couvrant les germes gram négatifs et les anaérobes a été démarrée, et une préparation du patient pour une chirurgie en urgence a été entamée mais le patient décédait d'une défaillance multiviscérale avec un syndrome de détresse respiratoire aigu, une heure après la réalisation du scanner abdominal.

**Figure 1:**
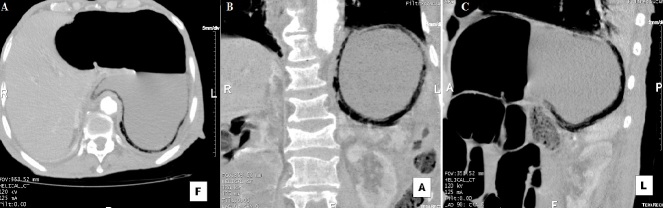
A) tomodensitométrie abdominale en coupe axiale en intensité de projection minimale (MinIP) mettant en évidence une pneumatose diffuse de la paroi gastrique sans pneumopéritoine; B) tomodensitométrie abdominale en reconstruction coronale en intensité de projection minimale (MinIP) mettant en évidence une pneumatose diffuse de la paroi gastrique sans pneumopéritoine; C) tomodensitométrie abdominale en reconstruction sagittale en intensité de projection minimale (MinIP) mettant en évidence une pneumatose diffuse de la paroi gastrique sans pneumopéritoine

